# Comparison of Non-Destructive Techniques for Technological Bridge Deflection Testing

**DOI:** 10.3390/ma13081908

**Published:** 2020-04-18

**Authors:** Jacek Kwiatkowski, Wojciech Anigacz, Damian Beben

**Affiliations:** 1 J&L Consulting Jacek Kwiatkowski, 45-594 Opole, Poland; j.kwiatkowski@jl-consulting.pl; 2 Faculty of Civil Engineering and Architecture, Opole University of Technology, 45-758 Opole, Poland; w.anigacz@po.edu.pl

**Keywords:** laser scanning, photogrammetry, tachymetry, bridge deflection, non-contact measurement

## Abstract

This paper presents a comparison and assessment of usefulness of various measuring techniques (terrestrial laser scanning (TLS), tachymetry, photogrammetry) applied to establish the behavior of a suspension bridge under different load scenarios. The applied techniques were examined on the bridge with a 165 m span. The tested structure works as the technological bridge for a belt conveyor linking a lime mine and cement plant. The testing range consisted of conducting the non-contact measuring of the bridge and cable displacements under dynamic loads (during the belt conveyor movement—normal service loads) and static loads (while stopped). Tachymetric surveys were carried out using a precise total station (to obtain the reference data). A Canon 750D digital camera was applied in the photogrammetry technique. FARO Focus 3D and Trimble TX8 scanners were employed for the TLS measuring. The obtained results are especially important for bridge inspectors and managers who can use the non-contact measurements of serviced structures.

## 1. Introduction

Generally, dynamic and static examinations are carried out to verify the bridge structure behavior versus design assumptions or to determine the actual load-carrying capacity of the bridge in connection to planned repair works. Conventional empirical experiments are normally conducted using gauges to measure accelerations, strains, displacements, and temperatures, as well as using wireless and geodesic techniques [1,2,3,4,5]. However, mounting the above-mentioned gauges requires the close contact with the examined construction. In some cases, there may be a problem with access to the testing objects, frequently needing the use of platforms, scaffolding, alpinists, cranes, etc. Consequently, this is the main motivation to examine and evaluate the chosen measuring techniques designed for non-contact observation on the bridge deflections.

Useful methods for observing the bridge deflections with interferometry, computer vision, digital image processing, a laser Doppler vibrometer, a Robotic Total Station, a GPS, and a video deflectometer are presented by Beben [6], Lee and Shinozuka [7], Meng et al. [8], Moschas and Stiros [9], Nassif et al. [10], Olaszek [11], Pan et al. [12], Pieraccini et al. [13], Pieraccini and Miccinesi [14], Psimulis and Stiros [15], Spencer et al. [16] and Zhang et al. [17]. Currently, some researchers are using new technology to monitor bridge behavior, such as for example the smartphone [18]. Most researchers, for example, Anigacz and Kokocinska-Pakiet [1], Schofield and Breanch [19], and Yu et al. [20], prefer a traditional way to do the object testing; i.e., they set a reference measuring grid both on the examined structure and outside. It requires signalizing the tested points on the structure. This way is completely appropriate but requires preparatory work in the field.

It should be emphasized that the latest achievements in measurement technologies allow obtaining a several million points set (called a point cloud) of the analyzed object. The point cloud can be obtained indirectly (photogrammetry) and directly (spatial terrestrial laser scanning (TLS)), as well by using both methods together. This allows receiving complete data about the object shape [21].

Jauregui et al. [22] reported an analysis of bridge displacements using the close-range photogrammetry method. The obtained results were compared to the total station readings and finite element analysis. Jiang et al. [23] presented the latest state-of-the-art on the close-range photogrammetry technique, including monitoring the bridge behavior and changes in its shape. However, the distance from the camera to the tested bridge was quite small. Yu et al. [20] described the latest measuring techniques and compared them to each other (Table 1). Nevertheless, the given data related to the TLS method is overestimated. This is because achieving precision up to 1 mm is practically impossible for the large-span bridges or limited to a short distance from the scanner to the bridge (in the case of small bridges). Yoon et al. [24] applied the TLS method to receive the crucial tunnel data.

In a typical survey with a conventional measurement instrument, the creation of a measuring grid is related to the transfer of potential errors to the observed points. Beshr [25] and Osada [26] proposed methods to reduce such errors. However, it should be emphasized that these problems did not appear in an analyzed situation. This is because the photogrammetric and TLS examinations were conducted from the same position. The connecting points of the individual scans constituted white reference spheres and measuring targets, as well as clearly visible bridge details.

The main aim of the research was to examine and evaluate the measurement possibilities for determining the bridge deflections under various load scenarios. The preliminary research for this bridge is given in [27]. Information about bridge deflections, obtained during normal operating conditions, is crucial from a structural safety point of view. In addition, the adequacy of the design principles may be assessed using data on the bridge behavior under load. Furthermore, in the case of old bridges, it is important to know that the admissible deflections of the load-bearing structure are not exceeded. One of the main purposes of the study was to minimize the preliminary activities on the examined bridge. Therefore, it was resolved to apply white reference spheres, surveying targets and well-visible bridge details as the measuring points. In the tachymetry method (as the reference method), several surveying targets were also attached on the transverse beams of the bridge. For this reason, two measuring positions were constantly maintained throughout the test. This allows a direct comparison of results of the test sessions conducted using various methods.

In the present paper, a suspension bridge with a 165 m span was tested under static and dynamic loading. In the tested bridge, typical deflection sensors could not be used because the main span of the bridge is located over a large river. Therefore, the photogrammetry and TLS methods were applied, and the obtained results were compared to a classic measuring technique (tachymetry—used as a reference method). Until now, most photogrammetric measurements were conducted at a relatively short distance from the tested structures. In this case, a greater distance from the camera to the bridge (ca. 120 m) was examined. The laser scanning results were also presented in the form of color-calibrated maps of deviations to present bridge deflections and the most strained elements (important for the bridge inspectors). This method of presenting results is new and highly useful because it supplies important information on deformation of the bridge elements. The most important results of the bridge deflections using applied methods were given and compared to each other and with appropriate values from the bridge standards. In addition, the benefits and drawbacks from each applied technique were also characterized.

## 2. Description of The Bridge Structure

**Figure 1:**
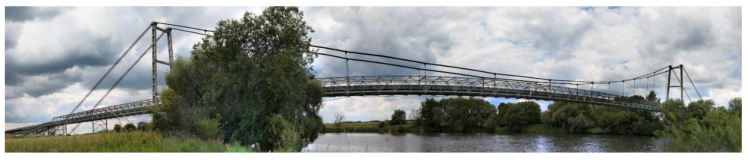
Side view of the bridge from the upper water side.

The examined object was a technological suspension bridge over the Odra River (Southern Poland) designed for joining a marl open-pit mine with a cement plant (Figure 1). Generally, the bridge includes five spans, the main span over the river (165 m) and four spans over the floodplain. The steel transport gallery (together with the belt conveyor) is suspended on two sets of carrying cables (six wires in each set) that are supported on two towers (in the form of portals) and anchored to special reinforced concrete blocks. Each wire in the set has a 48 mm diameter. The height of each tower is 26.50 m, and the total bridge length is 285.00 m. The floodplain sections of the bridge consist of self-supporting trusses with a 30.00 m span. To guarantee lateral stability of the bridge in the case of increased lateral wind action, the bottom parts of the bridge deck are also secured by two cables intersecting at one-third of the main bridge span. An orthotropic plate, made of a 5 mm thick flat sheet, and 50 mm channel bar cross stiffeners spaced at 0.50 m are placed directly on the longitudinal beams. The outermost segments of the river span are set on the lowest beam, concentrating the poles of the pylon. Connections between the hangers, the cables, and the structure of the bridge were designed by placing them in proper steel connectors. The bridge towers rest on a reinforced concrete foundation through the steel articulated bearings. The cross-section of the pole consists of two 550 mm I-sections, connected with batten plates and bridging comprising two I-sections, 500 mm each. The sets of wires are joined to the bridge tower using the head and bolted joints in such a way as to allow limited cable movement on the tower top. The greatest lateral movement on the bearings was anticipated at both sides of the bridge entrance, while tangential bearings were applied in the towers. 

The examined bridge was designed to carry the belt conveyor situated on the truss gallery (for limestone transport). The mass of the limestone output transported via the bridge was in the 900 to 1100 tons per hour range, and the velocity of the belt conveyor was about 7 km per hour. The bridge was loaded using evenly distributed loads (output of limestone). The quantity of limestone was automatically controlled by the feeder and was approximately constant. The bridge was recently refurbished by building a new composite (carbon fiber reinforced polymer) deck (instead of a steel orthotropic deck), changing a broken hanger, and eliminating tower cracks. Before the tests described here, no bridge damages were observed. Considering the national principles concerning evaluating bridge conditions, the structural rating was satisfactory.

## 3. Methodology of the Experimental Study

### 3.1. General Remarks

Generally, survey-engineering uses a wide scope of measuring techniques; therefore, it is important to select the most suitable one for bridge deflection testing, especially since the structure span is 165 m. Therefore, the critical determinants, affecting the choice of measuring technique, are the bridge shape, its parameters, and possible access to the bridge (for mounting the deflection sensors). Deflections of this bridge were also measured by Rabiega et al. [28] using a laser rangefinder. The results obtained were unsatisfactory due to the belt conveyor vibration, affecting the receiver and transmitter. 

The tested bridge was monitored using three independent measuring techniques: tachymetry, TLS, and photogrammetry. The measurement range of each applied method was the same. Thus, it can be assumed that those methods are mutually verified. It should be added that the measuring network with benchmarks was situated outside the testing bridge. In the analyzed case, the most important factors are the bridge length and the spatial nature of the bridge (the truss). The bridge length is important because the accuracy of determining the position of the measuring points falls with increasing sight length. It should be also added that before the main bridge testing, a preliminary examination of the selected methods was conducted to choose the most appropriate measuring shields, white spheres, and to determine the proper distances from instruments to the bridge. Additionally, various environmental conditions were also checked. The field testing was conducted after 40 years of bridge operation and a year after the bridge repair. Bridge deflections were recorded in three load scenarios:

(1) A: Static loading (the reference point for other test results, belt conveyor with limestone, without motion),
(2) B: Dynamic loading (the belt conveyor was running but it was empty, without limestone),
(3) C: Dynamic loading, i.e., normal operation (the belt conveyor with limestone was in motion).

Each load scenario (A, B, and C) lasted approximately 1 h, which gave 3 h for the full load cycle. In total, bridge testing took 12 h (four load cycles). The tachymetry method is a classical measurement method (one of the most accurate), and in this test, it was treated as the reference method. The bridge deflections were observed and compared to each other based on these techniques. To receive the reference stage for each applied method, the initial measurements were carried out under the static loading (without the limestone). All results obtained were referred to this stage. 

Environmental conditions can have a significant impact on the accuracy of the laser scanning and photogrammetric measurements. Producers of measuring equipment (laser scanners) introduce limitations to the control systems of these devices to prevent measurements when external conditions exceed their values. The most commonly considered factors are vibrations, temperature, and atmospheric pressure. The instrument’s internal software makes corrections automatically due to the refraction and curvature of the Earth. Some measuring instruments have the option of introducing additional factors, e.g., humidity. In summary, laser scanners have a zero and one environmental security system. This means that for the level of external factors set by the producer (or user), the instrument works correctly and after exceeding them, the instrument turns off. Factors affecting the measurement accuracy also include dust, fog, unfavorable lighting, and precipitation such as rain or snow. In addition, the impact of these factors may change over time. To check the influence of the selected environmental conditions, preliminary tests were conducted during the rain and high humidity (>80%). The obtained results were unsatisfactory due to the lack of repeatability of results (they deviated far above the standard error). Therefore, the authors tried to minimize the impact of external conditions on the accuracy of measurements by making them in the most favorable weather conditions. 

### 3.2. The Tachymetry Technique

**Figure 2:**
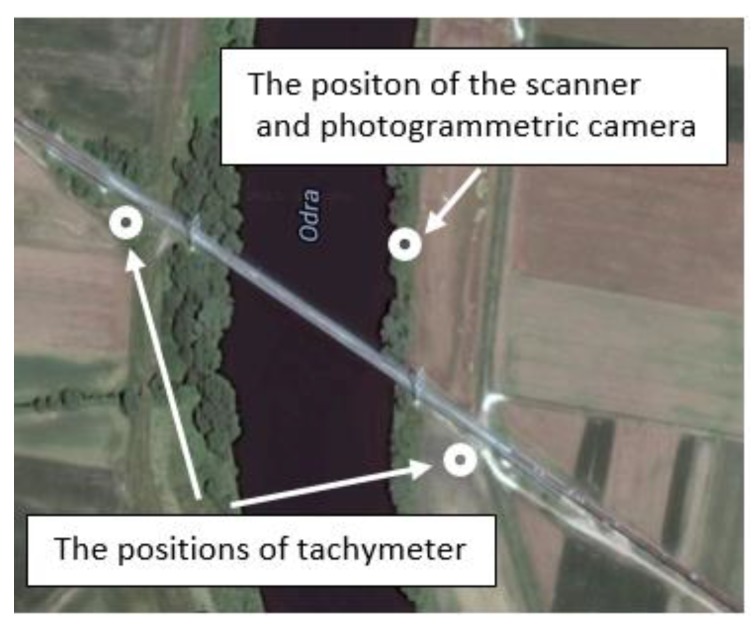
Top view on locations of total stations, laser scanner, and digital camera.

**Figure 3:**
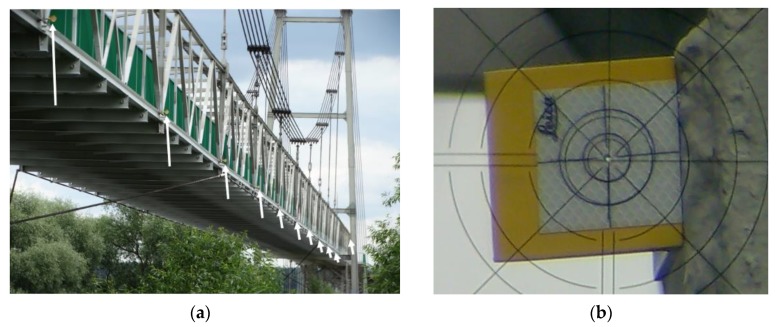
The examined bridge structure seen through the total station: (**a**) distribution of the measurement shields on the transport gallery, (**b**) view of the measurement shield through the telescope of the total station.

The tachymetry technique is related to creation of the spatial polar coordinates, i.e., vertical and horizontal angles, and the distances from the given reference points. Currently, total stations are theoretically available for measurements with a precision up to 1 mm and for measuring directions of 0.15 mgon (0.5’). However, considering practical experiences, the received accuracy is commonly significantly smaller, i.e., 1–5 mm for the distance measuring and 0.9–3 mgon (3–10’) for the direction measuring. In the analyzed example, to decrease the length of sight (the distance between the measuring points and the total station position), the positions of the total stations were located on both banks of the river (Figure 2). Thus, the length of sight did not exceed 100 m. Notwithstanding the significant discrepancy between the accuracy in the field and lab conditions, the tachymetry technique still seems to be the most precise way to determine the point location. This technique is distinguished by the discretion of measuring. A Leica TC2002 (Heerbrugg, Switzerland) total station was used for both tests. Several measuring points were selected (targets were placed on the transverse beams) for observing the bridge deflections (Figure 3). The bridge deflections were computed using the method proposed by Anigacz and Kokocinska-Pakiet [1].

The tachymetric measurements, as the most accurate measurements, were taken as the reference results for the other testing techniques. The received bridge deflection results have been confirmed by measuring from the second position of the total station. To distinguish the observed bridge deflections, the two-sided measuring shields were applied, which allowed measuring the distance from both sides of the bridge. The measuring shields were installed on the transverse beams of the bridge deck (Figure 3a). Thanks to this location of measurement points, it was possible to determine the deflection at 1/4, 1/2, and 3/4 of the span. A view through the telescope of the total station is presented in Figure 3b. The hole in the center of the shield has a 5 mm diameter. Based on multiple measurements of the total station-shield set used, a 1–2 mm real accuracy was obtained. 

### 3.3. The Photogrammetry Technique

The photogrammetry measuring method appeared almost simultaneously with the invention of photography. The last few decades have seen the development of digital photogrammetry, which has almost completely replaced analogue photogrammetry in engineering applications. The development of analytical methods has enabled the use of non-metric cameras in engineering applications. The non-metric cameras do not have to strictly meet the requirements for metric cameras, which makes them much cheaper. 

In the described case, a Canon 750D digital camera (Oita, Japan) with an 85 mm fixed focus lens was used to make the photograms. The camera was situated on a GigaPano turntable to automatically take the sequences of pictures. The distance from the camera to the tested bridge was 120 m. To verify the assumed method of photogrammetric measurements, a laboratory test was conducted. In the presented case, the lens distortion (with an 85 mm fixed focus lens) was determined based on a photo of 420 mm × 297 mm graph paper on which an ideal grid of squares was applied in AutoCAD (Version 23.0) [29].

**Figure 4:**
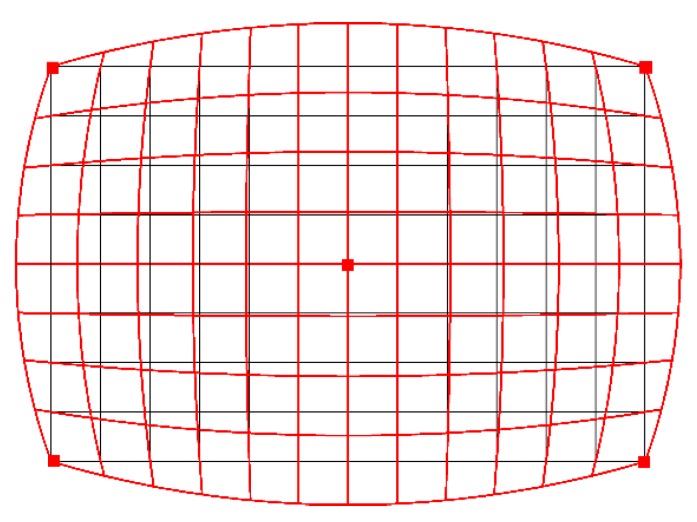
The barrel distortion (red) against the background of a standard grid of squares (black).

**Figure 5:**
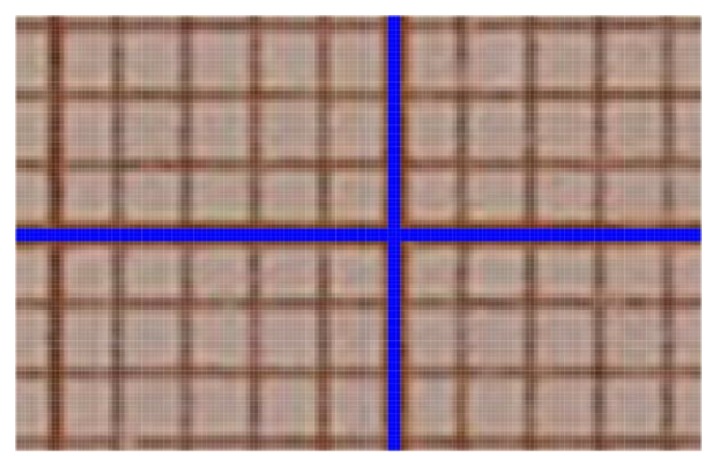
Camera matrix center on the background of the standard square grid (blue).

The key parameter for photogrammetric measurements is a barrel distortion (Figure 4). This figure shows the image of a square grid deformed by the barrel distortion (red) and the standard square grid (black). Figure 5 shows an image of the camera matrix center. The standard grid of squares (blue) almost perfectly coincides with the symmetry axes of the cropped graph paper. This means that for 1 pixel resolution, any lens errors are imperceptible.

**Figure 6:**
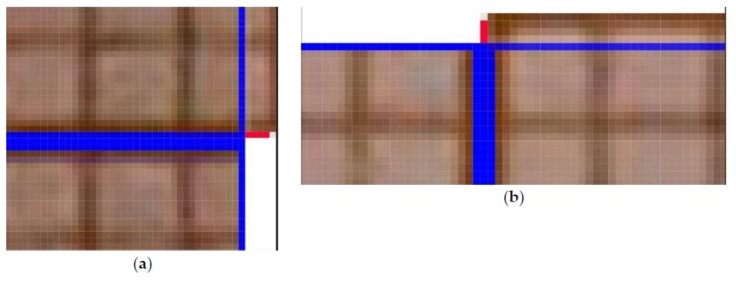
Maximum barrel distortion for: (**a**) the upper, (**b**) right edge of the frame, recorded in the middle of its length.

To verify the quality of the lens, differences (in pixels) between the theoretical (ideal) square grid (created in AutoCAD) and superimposed on the actual image of the square grid (obtained using the tested lens) were measured. The comparison was made in the middle of the outer sides of the frame, where the distortion should be the highest. The basis for determining the amount of distortion was taking a frontal photograph of a sheet of graph paper. Then, the corner image in the pixels was superimposed (shifting along the horizontal line) on the image halfway along the side. The number of pixels extending (in the vertical) beyond the theoretical frame (marked by the blue horizontal line—Figure 6) determines the level of barrel distortion. For the optical system used, the maximum distortion on the upper and lower edges of the frame was 4 pixels (Figure 6a), which means the percentage deformation (2 × 4)/4000 = 0.002, i.e., 0.2%. In other words, the length of the vertical line in the center of the frame is 4008 pixels instead of 4000 pixels, which gives an error of 0.2% ((4008–4000)/4000 = 0.002). One square corresponds to one pixel (Figure 6a). In the analyzed case, the Canon 750D camera matrix has a 22.3 × 14.9 mm physical size and 6000 × 4000 in pixels. The same was done for the shorter edges of the frame, i.e., shifting the corner image along the blue vertical line to the middle of the edge length shows the level of barrel distortion on the short side of the frame. The maximum distortion was 3 pixels (Figure 6b), which means that the percentage deformation is (2 × 3)/6000 = 0.001, i.e., 0.1%. The length of the horizontal line in the center of the frame instead of is 6006 pixels, 6000 pixels, which gives an error of 0.1% ((6006−6000)/6000 = 0.001).

In summary of the lab tests, the registered deformation of the camera-lens system used allows for computerized adjustment with an accuracy of one pixel (Figure 6). Only the central part of the frame (2000/1333 pixels), constituting 1/9 of the entire surface of the frame, was used for the measurement. This is due to use of stitching covering 1/3 of the frame. For the 2000/1333 pixel frame field used, the distortion does not exceed 1 pixel, which is within the interpretational error limit. Therefore, for the expected measurement accuracy and the inability to register deformations in the central part with an accuracy below 1 pixel, it is justified to abandon the calibration procedure.

The AutoPano Giga 4.0 program was applied to process the photograms. The Photoshop CC program (Version 19.0) was applied to calibrate the produced photogram. This was done using a linear rescaling to determine the size of a single pixel.

**Figure 7:**
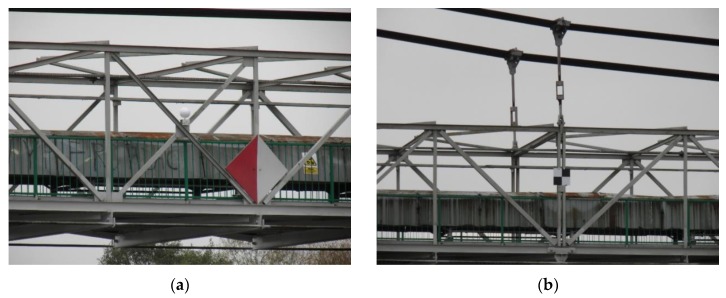
The supplementary measuring components fixed on the structure: (**a**) sphere, (**b**) measuring square shield (checker).

To receive the bridge deflections at the stage of the dynamic tests, the digital camera was positioned so that it could take a series of photographs at an assumed time, e.g., five photograms per second. As a result, the bridge deflection distributions were measured. To rescale the bridge photograms to the fixed pixel size, the distinctive bridge elements were taken (i.e., the distance between transverse beams of the bridge deck), which were tested earlier using the total station (Figure 3a). The supplementary measurement components, i.e., white reference spheres (Figure 7a) and measuring square shields (Figure 7b), were mounted on the bridge. The photogrammetry technique is characterized by various benefits, such as short measuring time, spatial visualization, and relatively low-cost equipment (digital camera). The results of photogrammetric measurements are dependent mainly on the following determinants:

(1) Digital camera matrix resolution,
(2) Optical system quality (a decrease of the spherical deviation, resolution),
(3) Program capacity to process the photograms, e.g., joining the individual images.

### 3.4. The TLS Technique

The TLS technique permits receiving a quasi-constant, three-dimensional presentation of the observed objects. The precision of the measuring effects depends mainly on the sight length, i.e., the distances between the elements of the tested object and the scanner. The number of the TLS applications is growing rapidly [21,22,30]. TLS measurements are related to determining the distance based on a difference between the reflected and emitted laser beam. Data on the obtained distances and the reflected signals are transformed into coordinates in real time. The operation principles are described in detail by Rodriguez [31].

**Figure 8:**
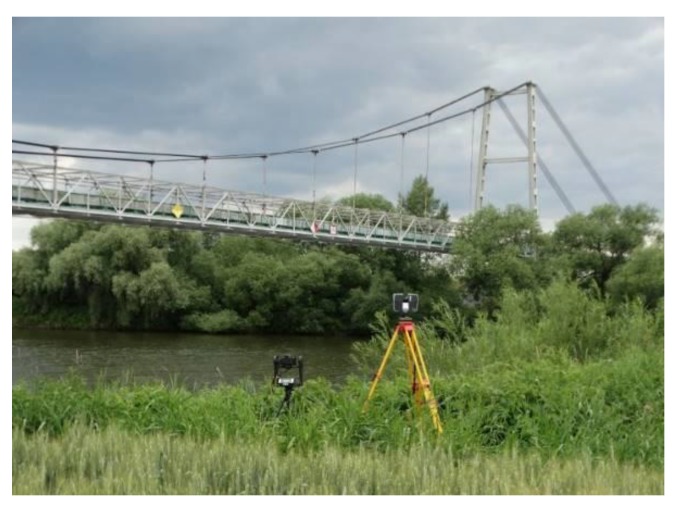
Side view of the bridge measurement sets for laser scanning and photogrammetry.

FARO Focus 3D (Lake Mary, FL, USA) and Trimble TX8 scanners (Sunnyvale, CA, USA) (Figure 8) were applied for the bridge deflection testing. These scanners measure almost 1 million points per second and allow building a cloud consisting of even a billion points. The manufacturer declares that the measurement precision of the FARO Focus 3D scanner is on the level of ±2 mm, while the angular resolution is equal to 0.16 mrad. The maximum scanning range is up to 130 m. The maximum scanning range of the Trimble TX8 scanner is 120 m, but in favorable conditions, it may even extend to 340 m. The laser wavelength is 1.5 μm, and the scanning frequency is 1 MHz. The declared measurement accuracy is ±2 mm, and the angular resolution is 0.07 mrad. A comparison of catalogue data shows that the Trimble TX8 scanner has twice the angular resolution as the FARO Focus 3D scanner. Anigacz et al. [32] applied the FARO laser scanner during the first attempt to check the applicability of the TLS technology to bridge deflection testing. However, the results indicate that the FARO laser scanner used did not allow for obtaining accurate and satisfactory data. The quality of the scanning results of the bridge deflections was close to the maximum range of the scanner (120 m). For this reason, the quality of scans (density of the points) was too weak to obtain the bridge deflections with satisfactory accuracy.

The Trimble TX8 scanner allows obtaining the point clouds for the unloaded and loaded state clouds with a 5.7 mm resolution at a 30 m distance. This gave about 500 million measurement points for a single scan. The unloaded state was registered on five measuring spheres to the loaded state with accuracy under 1 mm using Trimble RealWorks Advanced Plant software version 10.2 [33]. Visualization of the deviation cloud between the unloaded and loaded state was obtained through an authoritative optimization approach that was then used for comparative analysis in Trimble RealWorks Advanced Plant 10.2. The software permits for the autointerpolation of the center of the measuring shields and spheres, which were also used in the photogrammetry technique (Figure 7).

It is important that measuring scanning sessions were compatible with each other; therefore, succeeding scans should be matched to selected bridge points. This approach significantly reduces the measurement time on the structure.

When TLS is used, the compared point clouds are recorded in a common coordinate system, or both are georeferenced if a global reference system exists. In the analyzed case, one point cloud represents the bridge state without load and the other cloud represents the state with a static load. There are two cases of point identification, i.e., characteristic points of the structure and points signaled by markers recognized by the software (e.g., checker, spheres). It should be emphasized that much better accuracy in determining the location of points is obtained when they are signaled (as in the tested bridge).

In this case, the results of laser scanning were additionally presented in the form of color deviation maps. The generation of deviation maps consists of comparing a georeferenced point cloud with a 3D model. Based on differences in the distance between the 3D model and the point cloud, the system allows you to give colors to the cloud with a given RGB value depending on the reference distance (i.e., the surface of the 3D model). In the analyzed case, navy blue was used for 0–4 mm deformations, green-yellow was used for 24–32 mm deformations, and red was used for 40–50 mm deformations. The color-defined deviation maps can be indicated by the operator performing the study. 

## 4. Analysis of Bridge Deflections

From a practical point of view, it is important to know the maximum bridge deflections. Therefore, the authors decided to analyze the bridge behavior at selected points located at 1/4, 1/2, and 3/4 of the main bridge span. In total, 10 node points situated on the bridge deck were analyzed during the measurements (Figure 3a). In addition, two bridge towers were also observed. It should be emphasized that measurements using the tachymetry technique (the reference method) were conducted over a longer period of time. Nevertheless, the authors tried to record the data at a similar time as for other methods. The possible differences in the bridge deflections can be related to the insufficient synchronization of tests carried out with various techniques. 

**Figure 9:**
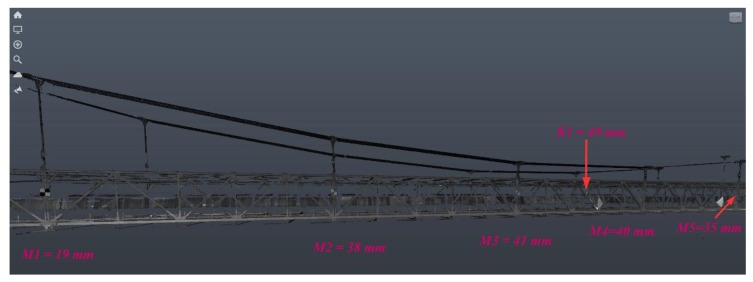
Bridge deflections under static loading (A) in chosen points received from the TLS technique (Trimble scanner).

Figure 9 presents an example of the obtained bridge scans. The maximum deflection results for the selected bridge points are presented in Table 2. Other maximum deflections in each measurement series were similar to those presented in Table 2 (the maximum difference did not exceed 1–3% for considered load scenarios). Therefore, it can be assumed that the results obtained were relatively convergent in individual load cycles.

The obtained results also show the bridge deflections for various load scenarios (A, B, and C), and it is easy to observe the differences obtained between applied measuring techniques. The presented results were calculated in relation to the dead load of the bridge (reference stage: static loading without the limestone). Maximum bridge deflections were 60 mm and 49 mm for the photogrammetry and TLS (Trimble scanner), respectively, which means that the TLS results are quite consistent with the reference method (tachymetry); in contrast, the maximum bridge displacements obtained from the photogrammetry technique are higher by 18–20% than those obtained from the tachymetry and TLS techniques. These differences are related to the pixel size of the applied digital camera (4 mm) in the photogrammetry technique. Generally, the maximum deflections were observed in the middle of the main span of the bridge. The bridge deflections at other points (at 1/4 and 3/4 of the bridge span) were much lower. 

The obtained results expressly confirmed that the bridge deflections under the static loading (load scenario A) were greater than those received under dynamic loading (load scenario C). The obtained disparities differed depending on the bridge measuring points (17–37%). This is due to load scenario A, where the load (limestone) can be considered as a linear load, and it was positioned stationary on the bridge. The measurement under this load lasted at least 1 h. The bridge deflections according to the load scenario B (dynamic loading without limestone) were at a low level.

Generally, the bridge deflections received from the TLS and tachymetry techniques under the static loading (A) are quite close to each other (Table 2). The TLS method for the bridge deflection measurements requires application of an adequate scanner, i.e., Trimble TX8. The results are convergent with the reference: the tachymetry method. Using the FARO Focus 3D scanner was less precise. It was the result of the too-large distance between the apparatus and the bridge and too-small angular resolution of a laser scanner (point density). In addition, the fact that the suspension bridge has an openwork structure also affects poor results (scans of larger density than ones obtained from the FARO scanner are required). To verify the test, the same scanner (FARO) was used to conduct inventory work on a historic (the oldest in Europe) suspension cast-iron bridge in Ozimek on the Mala Panew river (Poland). The tests were conducted at a much shorter distance between the scanner and the bridge, i.e., 40 m. The recorded point cloud density allowed identifying the elements (details) of the bridge, and fully satisfactory bridge inventory results were obtained [32].

**Figure 10:**
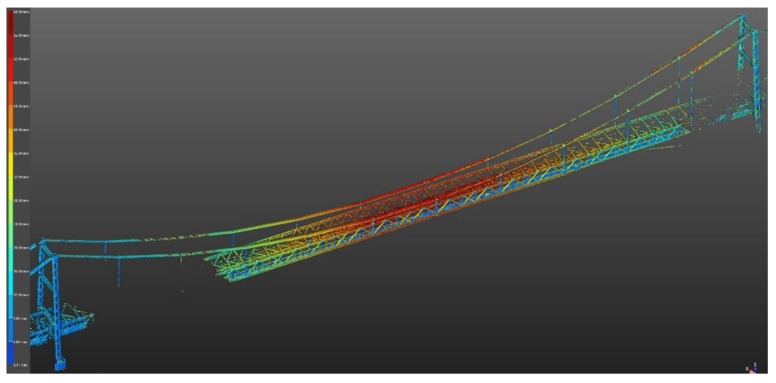
Bridge displacements under load scenario A shown by means of color deviation maps (Trimble TX8 scanner).

**Figure 11:**
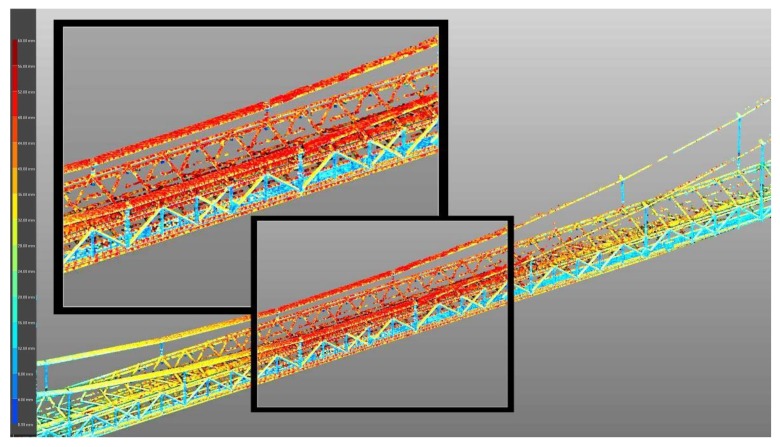
View of bridge deflection details (in colors) using a Trimble TX8 scanner.

Additionally, the TLS results can be presented using a calibrated map of colors (Figure 10). This method of presenting the TLS result is helpful for determining the most strenuous bridge elements. In addition, it indicates the bridge elements (in colors) where the deformations can be exceeded due to damages (which are not visible from the bridge deck). In general, incorrect behavior of some bridge elements can be detected. With this information, the bridge inspector can pay more attention to these elements during inspections. Red indicates the largest deflections and navy blue indicates the smallest. Maximum deflections of 49 mm were obtained in the middle of the main bridge span (they were recorded in suspension cables and the upper parts of the transport gallery). Additionally, the deformations of the bridge pylons are small (Figure 10). However, due to the resolution of a single screen, Figure 10 presents an indicative image that shows the tendency of displacements of the entire bridge structure. For a more accurate reading of displacements, in software working with point clouds, it is necessary to enlarge the given bridge element (Figure 11) or to measure the location of the same point in two clouds, one representing different states of the structure and one that is georeferenced. Figure 11 shows the details of the bridge deflections under the static loads (load scenario A) in the middle part of the structure obtained using a Trimble TX8 scanner. This also presents the highest deflection of bridge elements (red—cables and upper elements of the truss). 

Considering the TLS measuring sessions and the TLS results analysis, it is not possible to distinguish the bridge deflections under the dynamic loading. This is related to relatively rapidly changing bridge deflections in relation to the rotation speed of the laser scanner. Further research is needed on this topic. The authors believe that using the “in-line scanning” function can give interesting results. Then, multiple one-line scans (for example, near the middle of the bridge span) may allow determining the bridge deflections under the dynamic loading.

**Figure 12:**
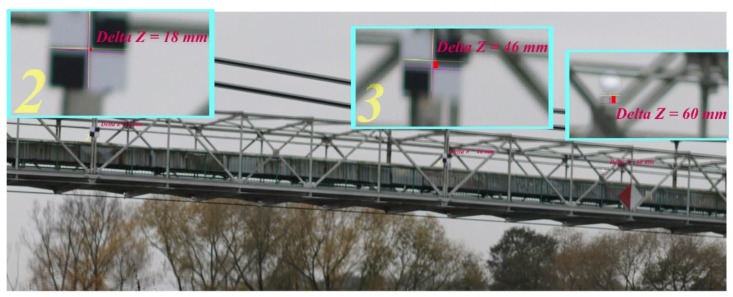
Bridge deflections under static loading (A) obtained from the photogrammetry.

Figure 12 shows the chosen bridge deflections received from the photogrammetry technique under the static loading (load scenario A). Well-identified points of the bridge structure were chosen for the measurements. Generally, the processing of the received photogrammetric results was based on overlapping the subsequent photograms (from the various load scenarios). For example, the first photogram came from the static loading scenario (without the limestone), and the next photogram displays two photograms superimposed (with and without the loads). On the images superimposed (presented in the small windows), one pixel has been rescaled to 10 mm. Next, the number of pixels of the tested bridge components moving to each other was determined (similar to in the lab tests mentioned earlier).

**Figure 13:**
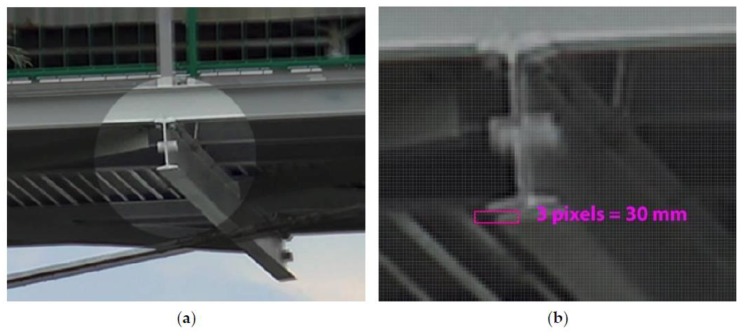
The analyzed detail in 3/4 of the bridge span: (**a**) without load, (**b**) images superimposed (without and with load) showing the scale of the bridge deflection under dynamic loads (load scenario C).

The deflection of the bridge section (crossbeam) under the dynamic loading (load scenario C) using the photogrammetry technique is presented in Figure 13. The first photogram (Figure 13a) was made when the belt conveyor was stopped (without the load), and the next one (Figure 13b) presents the image with two photograms superimposed (static loading and dynamic with the limestone (influence of the service dynamic loads)). As during the static loading (A), Figure 13b presents the superimposed images in which one pixel means 10 mm. As a result, the change in the bridge deflections under load scenario C (effect of normal bridge operation) was obtained. The results obtained indicate that the long distance from the digital camera to the bridge (ca. 120 m) did not adversely affect the quality of the bridge deflection readings. It is important to choose the appropriate camera focus, which means that the pixel size should be as large as possible, e.g., 2 mm. A larger scale (larger camera’s focus) of the photograms may permit obtaining detailed data on the observed bridge elements, e.g., cracks, corrosion, excessive distortions, etc. This can be useful for bridge managers and inspectors.

The obtained measurement errors in the case of the tested bridge are consistent with those reported by the manufacturers of the applied instruments. They were confirmed at reference points for multiple measurements of the measured quantity. The real measurement errors for the tested bridge were as follows: 2 mm for the tachymeter, 1 pixel (4 mm) for photogrammetry, and 2 mm for the TLS method.

The tested structure constitutes the technological bridge (without any specific requirements); therefore, the obtained results were compared to the regulations for railway and road bridges. The deflection limit of the steel railway bridges, in accordance with the bridge standard EN 1991-2 Eurocode [34], was calculated using the formula *l*/15*v*−400, where *v* is the maximum speed and *l* is the bridge span. The measured maximum deflection under the static loading in the middle of the main span is in the upper limit of permissible displacements (56 mm). However, considering the admissible deflections provided for the steel road bridges (PN-82/S-10052 [35]), the measured maximum test deflections are considerably below the admissible value (*l*/500 = 330 mm). To recap, the tested bridge (after repair) fulfills requirements for the steel road bridges. 

It would be best to measure the bridge deflections with a few total stations and digital cameras at the same time. Then, it would be possible to control the selected points using various techniques. As a result, accurate bridge deflections would be achieved. This approach would allow a complete measuring synchronization, but this way is practically impossible to conduct. This is due to the need to monitor a dozen points on the bridge at the same time. The total station and scanner need some time to take the measurements. The full synchronization is possible only for photogrammetry method and requires using several cameras that would be simultaneously activated by radio waves.

## 5. Conclusions

Considering the data gained from the bridge deflection testing under various load situations, carried out with using the TLS, photogrammetry, and tachymetry technologies, these final conclusions can be formulated:

(1) Tachymetry and photogrammetry techniques allow obtaining the bridge deflection under the static and dynamic load scenarios. However, the photogrammetry method gave overestimated deflections by 18% compared to the reference method: tachymetry. The long distance from the digital camera to the bridge (ca. 120 m) did not adversely affect the obtained deflection results.
(2) At the present time, the TLS technique enables obtaining bridge deflections during the static loading with almost identical precision as for tachymetry (reference method). Nevertheless, more experimental research is required for the TLS technology, especially for determining bridge behavior under rapidly changing dynamic loads.
(3) The elaborated TLS technology is particularly useful for topologically complex structures such as truss bridges. Automatically generating colors using calibrated deviation maps of the differences between scans (e.g., without and with load) allows selecting the points and elements of the bridge to which inspectors should pay more attention. Therefore, this method can also be helpful in bridge management.
(4) The greatest bridge deflection amounted to ca. cca 49 mm and it was identified in the center of the bridge span. This deflection was measured using the reference method (tachymetry) and photogrammetry. The highest bridge deflections, generated during the normal operation (load scenario C), should be defined as comparatively small in comparison to static loading (load scenario A). The bridge deflections received from the static loading are greater than those caused by dynamic loading. The received maximum deflection was considerably smaller than the permissible value for the road bridges; simultaneously, it was in the upper limit of permissible deflection in the regulations for the railway bridges.
(5) Taking into account all the analyzed measuring technologies, the TLS is the costliest; however, it allows presenting the data using colored maps. The photogrammetry technique permits conducting the testing of the bridges and other structures relatively cheaply and with satisfactory accuracy. For static loading, the TLS technique is better than the photogrammetry method; however, in the case of dynamic loading, it is the opposite. It would be best if both methods complement each other.

## Figures and Tables

**Table 1:** A set of displacement measuring techniques.

Measuring Techniques	Accuracy	Test Type	Bridge Class	Test Costs
Inclination	<cm	Static/dynamic	large rigid frame	low
Leveling	<mm	Static	no limits	low
Interferometry	<mm	Static/dynamic	no limits	expensive
Global Positioning System	cm	Static/dynamic	large span	average
Terrestrial laser scanning	<mm	Static	medium or small span	expensive
Connection pipe	<mm	Static	no limits	expensive
Electro-optical imaging	mm/cm	Static/dynamic	large span	average *
Computer vision	mm	Static/dynamic	large span	average
Tachymetric	mm	Static/dynamic	no limits	low

* Depends on used instruments.

**Table 2:** Results of the maximum recorded bridge deflections (in mm).

Measuring Technique	Load Scenarios	Bridge Deflection at the Span of:		
		1/4	1/2	3/4
Tachymetry (reference method)	A	24.0	49.1	37.7
	B	0.3	2.1	1.7
	C	19.7	35.4	24.1
Photogrammetry	A	18	60	46
	B	0	3	2
	C	15	43	30
TLS (FARO Focus)	A	^a^	^a^	^a^
TLS (Trimble TX8)	A	19	49	38
	B	^b^	^b^	^b^
	C	^b^	^b^	^b^

^a^ Former TLS results [32] were considered uncertain (the quality of point cloud was weak); ^b^ bridge deflections under dynamic loading are not possible to extract at this stage (the next research are still required); A, B, and C mean the load scenarios according to static loading (with limestone), dynamic loading (without the limestone), and dynamic loading (with limestone), respectively.
